# Editorial: Heterogeneity of clinical phenotypes in type 1 diabetes and of beta cell deterioration in type 1 diabetes

**DOI:** 10.3389/fendo.2022.1120555

**Published:** 2023-01-06

**Authors:** Xia Li, Mei Zhang, Jinhua Yan, Zhenqi Liu

**Affiliations:** ^1^National Clinical Research Center for Metabolic Diseases, Key Laboratory of Diabetes Immunology (Central South University), Ministry of Education, and Department of Metabolism and Endocrinology, The Second Xiangya Hospital of Central South University, Changsha, Hunan, China; ^2^Department of Endocrinology, the First Affiliated Hospital of Nanjing Medical University, Nanjing, China; ^3^Department of Endocrinology and Metabolism, the Third Affiliated Hospital of Sun Yat-sen University, Guangdong Provincial Key Laboratory of Diabetology, Guangzhou, China; ^4^Division of Endocrinology and Metabolism, Department of Medicine, University of Virginia Health System, Charlottesville, VA, United States

**Keywords:** type 1 diabetes, heterogeneity, precision medicine, disease management, omic analyses

Precision medicine is of particular importance in diabetes as it is a highly heterogeneous syndrome (1) that contains genetic, environmental, behavioral and other etiological and pathophysiological components. Information garnered through many large multicenter cohort studies, multi-integration of bio-information obtained using genomics, epigenomics, proteomics and metabolomics approaches, and the utilization of data-driven classification strategies have drastically altered our understanding of diabetes, especially type 2 diabetes (T2DM), heterogeneity. Indeed, patients with T2DM can be stratified using the clustering method into five subgroups with distinctive characteristics, which has been replicated in cohorts with diverse backgrounds, and this stratification has been proved to be effective for complication prevention and treatment tailoring (2–4). The application of machine learning methods in diabetes classification in more integrative data sources and a soft-clustering strategy to sub-classify patients using genome-wide association studies (GWAS) (5) have all been proved useful. A continuous classification for diabetes has been attempted using the “palette model” concept (6). Newly diagnosed patients with T2DM could be classified into four distinctive subgroups and one subgroup with mixture characteristics (7). On the other hand, while studies in precision medicine have been thriving in T2DM, our understanding of heterogeneity in type 1 diabetes (T1DM) remains limited.

With an autoimmune pathogenesis and the insulin dependency nature, T1DM has traditionally been perceived as a more homogeneous entity compared to T2DM. However, recent evidence suggests that T1DM is also a heterogeneous disease and significance advances have been made in precision prediction, prevention, diagnosis, and treatment in T1DM (8). Mounting evidence has confirmed the importance of precision medicine in T1DM. An insightful understanding of the etiology of T1DM revealed the role of novel omics biomarkers in addition to genetics and immunity (9). Clinical parameters such as islet autoantibodies, age of disease onset, body mass index (BMI), C-peptide, as well as genetic factors such as human leukocyte antigen (HLA) and genetic risk score (GRS) were utilized in T1DM sub-phenotyping using hypothesis-driven and data-driven approaches (10–12). Continuous glucose monitoring (CGM) and artificial intelligence have been used to direct personalized insulin use and glucose management (13). Different patterns of beta cell function decline have also been observed in patients with T1DM (14). These new developments have critically advanced our understanding of the underlying pathophysiology and molecular mechanisms of T1DM and are instrumental in personalized management of T1DM.

The current issue of Frontier in Endocrinology summarizes recent advances in heterogeneity in T1DM with a special emphasis in beta cell function deterioration. Amongst the articles included, three clinical study articles provided new insights into diagnosis, phenotyping, and treatment of T1DM. The comparative effectiveness of continuous subcutaneous insulin infusion (CSII) therapy versus multiple daily insulin injection (MDI) in persons with T1DM was evaluated using real-world data, and the authors found that CSII therapy was safer and more effective for the management of Chinese T1DM patients, resulting a lower HbA1c and better CGM-derived glycemic metrics without an improvement in hypoglycemia risks when compared with MDI therapy. In the study examining the association between metabolic syndrome (MetS) and glycemic variability in patients with T1DM using CGM systems, it was found that MetS was associated with worse glycemic control with high glucose variability (GV) and HbA1c levels. Thereby, efforts should be made to manage MetS components in patients with T1DM in order to achieve a better glycemic control. Finally, in an effort to refine the diagnosis of T1DM partial remission (PR), a new formula based on insulin dose and glycated albumin (GA) proved to be more precise and the cut-off value of C-peptide levels for representing transient “recovery” of beta cell function varies based on age.

Two studies performed omics detection to explore novel biomarkers for T1DM. One study was conducted in 1,005 islet autoantibody positive T1DM patients and 1,257 healthy controls to investigate the role of Single Nucleotide Polymorphisms (SNPs) in N6-methyladenosine (m6A) regulators in T1DM. Intronic variants in PRRC2A and YTHDC2 were found to be associated with T1DM tendency in a Chinese Han population, which indicates a role of m6A regulators and their intronic SNPs in the pathogenesis of T1DM. The other study on proteome profiling was performed in plasma samples and plasma-derived extracellular vesicles from pre-diabetic non-obese diabetic (NOD) mice and revealed new and potentially endotype specific biomarkers for T1DM occurrence.

The rest two articles are reviews on β-cell protection and therapy for latent autoimmune diabetes in adult (LADA) and the T cell immunometabolism and therapeutic implications in T1DM, respectively. In the first review, the authors described the natural history of LADA and summarized the factors related to β-cell function in LADA patients. They also evaluated the impact of current anti-hyperglycemic therapies on islet β-cell protection in LADA patients and discussed the opportunities and challenges for disease treatment in the future. In a mini review, the authors provided an overview of metabolic reprogramming of T cells, summarized the key metabolic pathways involved in the disease progression, and proposed the potential options for β-cell protection in T1DM.

Precision medicine in T1DM is currently a hot research topic given the limited understanding of disease pathophysiology and high disease heterogeneity. This special issue of Frontier in Endocrinology aims to provide an in-depth and integrative understanding of precision medicine for T1DM, reveal novel biomarkers related to diabetes sub-classification and disease progression, and explore new ways to diagnose, predict, prevent and manage T1DM. Precision prediction, diagnosis, treatment, and prevention will enable us to care patients with T1D in a person-centered manner, thus more effective and safer, in the near future.

## Author contributions

All authors listed have made a substantial, direct and intellectual contribution to the work, and approved for its publishment.

## References

[1] Chung WK Erion K Florez JC Hattersley AT Hivert MF Lee CG. Precision medicine in diabetes: A consensus report from the American diabetes association (ADA) and the European association for the study of diabetes (EASD). Diabetes Care (2020) 43(7):1617–35. doi: 10.2337/dci20-0022 PMC7305007 32561617

[2] Ahlqvist E Storm P Käräjämäki A Martinell M Dorkhan M Carlsson A. Novel subgroups of adult-onset diabetes and their association with outcomes: a data-driven cluster analysis of six variables. Lancet Diabetes Endocrinol (2018) 6(5):361–9. doi: 10.1016/S2213-8587(18)30051-2 29503172

[3] Dennis JM Shields BM Henley WE Jones AG Hattersley AT. Disease progression and treatment response in data-driven subgroups of type 2 diabetes compared with models based on simple clinical features: an analysis using clinical trial data. Lancet Diabetes Endocrinol (2019) 7(6):442–51. doi: 10.1016/S2213-8587(19)30087-7 PMC6520497 31047901

[4] Zaharia OP Strassburger K Strom A Bönhof GJ Karusheva Y Antoniou S. Risk of diabetes-associated diseases in subgroups of patients with recent-onset diabetes: a 5-year follow-up study. Lancet Diabetes Endocrinol (2019) 7(9):684–94. doi: 10.1016/S2213-8587(19)30187-1 31345776

[5] Udler MS Kim J von Grotthuss M Bonàs-Guarch S Cole JB Chiou J. Type 2 diabetes genetic loci informed by multi-trait associations point to disease mechanisms and subtypes: A soft clustering analysis. PloS Med (2018) 15(9):e1002654. doi: 10.1371/journal.pmed.1002654 30240442 PMC6150463

[6] Pearson ER. Type 2 diabetes: a multifaceted disease. Diabetologia (2019) 62(7):1107–12. doi: 10.1007/s00125-019-4909-y PMC6560016 31161345

[7] Wesolowska-Andersen A Brorsson CA Bizzotto R Mari A Tura A Koivula R. Four groups of type 2 diabetes contribute to the etiological and clinical heterogeneity in newly diagnosed individuals: An IMI DIRECT study. Cell Rep Med (2022) 3(1):100477. doi: 10.1016/j.xcrm.2021.100477 35106505 PMC8784706

[8] Carr ALJ Evans-Molina C Oram RA. Precision medicine in type 1 diabetes. Diabetologia (2022) 65(11):1854–66. doi: 10.1007/s00125-022-05778-3 PMC9522741 35994083

[9] Xhonneux LP Knight O Lernmark Å Bonifacio E Hagopian WA Rewers MJ. Transcriptional networks in at-risk individuals identify signatures of type 1 diabetes progression. Sci Transl Med (2021) 13(587):eabd5666. doi: 10.1126/scitranslmed.abd5666 33790023 PMC8447843

[10] Lynam A McDonald T Hill A Dennis J Oram R Pearson E. Development and validation of multivariable clinical diagnostic models to identify type 1 diabetes requiring rapid insulin therapy in adults aged 18-50 years. BMJ Open (2019) 9(9):e031586. doi: 10.1136/bmjopen-2019-031586 PMC6773323 31558459

[11] Shields BM Peters JL Cooper C Lowe J Knight BA Powell RJ. Can clinical features be used to differentiate type 1 from type 2 diabetes? a systematic review of the literature. BMJ Open (2015) 5(11):e009088. doi: 10.1136/bmjopen-2015-009088 PMC4636628 26525723

[12] Holt RIG DeVries JH Hess-Fischl A Hirsch IB Kirkman MS Klupa T. The management of type 1 diabetes in adults. a consensus report by the American diabetes association (ADA) and the European association for the study of diabetes (EASD). Diabetes Care (2021) 44(11):2589–625. doi: 10.2337/dci21-0043 34593612

[13] Oh J Kim JH Park HD. Clinical utility and cross-reactivity of insulin and c-peptide assays by the lumipulse G1200 system. Ann Lab Med (2018) 38(6):530–7. doi: 10.3343/alm.2018.38.6.530 PMC6056384 30027696

[14] Shi M Xie Y Tang R Zhong T Zhou Z Li X. Three-phasic pattern of c-peptide decline in type 1 diabetes patients with partial remission. Diabetes Metab Res Rev (2021) 37(8):e3461. doi: 10.1002/dmrr.3461 33928751

